# *Thiothrix* and *Sulfurovum* genera dominate bacterial mats in Slovak cold sulfur springs

**DOI:** 10.1186/s40793-023-00527-4

**Published:** 2023-09-20

**Authors:** Lea Nosalova, Chahrazed Mekadim, Jakub Mrazek, Peter Pristas

**Affiliations:** 1 Department of Microbiology, Institute of Biology and Ecology, Faculty of Science, Pavol Jozef Safarik University in Kosice, Srobarova 2, Kosice, 041 54 Slovakia; 2 Laboratory of Anaerobic Microbiology, Institute of Animal Physiology and Genetics, Czech Academy of Sciences, Videnska, Prague, 1083, 14220 Czech Republic; 3 Institute of Animal Physiology, Centre of Biosciences, Slovak Academy of Sciences, Soltesovej 4-6, Kosice, 040 01 Slovakia

**Keywords:** Sulfur-oxidizing bacteria, Sulfur springs, Microbiota, Slovakia

## Abstract

Microbiota of sulfur-rich environments has been extensively studied due to the biotechnological potential of sulfur bacteria, or as a model of ancient life. Cold terrestrial sulfur springs are less studied compared to sulfur-oxidizing microbiota of hydrothermal vents, volcanic environments, or soda lakes. Despite that, several studies suggested that sulfur springs harbor diverse microbial communities because of the unique geochemical conditions of upwelling waters. In this study, the microbiota of five terrestrial sulfur springs was examined using a 16 S rRNA gene sequencing. The clear dominance of the *Proteobacteria* and *Campylobacterota* phyla of cold sulfur springs microbiota was observed. Contrary to that, the microbiota of the hot sulfur spring was dominated by the *Aquificota* and *Firmicutes* phylum respectively. Sulfur-oxidizing genera constituted a dominant part of the microbial populations with the *Thiothrix* and *Sulfurovum* genera identified as the core microbiota of cold sulfur terrestrial springs in Slovakia. Additionally, the study emphasizes that sulfur springs in Slovakia support unique, poorly characterized bacterial communities of sulfur-oxidizing bacteria.

## Background

The diversity of bacteria living in various environments is dictated by ecological factors [1]. Due to physiochemical conditions, sulfur spring ecosystems sustain the growth of a narrow range of bacteria only, which also includes sulfur-oxidizers [2]. A complex relationship exists between the sulfur-oxidizing bacteria and the environment they inhabit, as they interact intimately with sulfur species available in their surroundings [3]. Therefore, the type of environment suitable for sulfur-oxidizing bacteria is restricted by the availability of sulfur compounds with certain redox states [4]. In these environments, reduced sulfur compounds generated by microorganisms that reduce sulfur species provide an energy source for sulfur-oxidizing bacteria, although a larger fraction of reduced sulfur compounds may come from geological processes [5].

Sulfur-oxidizing microbial communities have been of special interest to microbiologists for more than a hundred years since the studies on *Beggiatoa* and the discovery of the chemolithotrophy by Winogradsky [6]. Despite that, their occurrence and relationship to geochemical conditions are still poorly understood [7]. Microbial sulfur oxidation and reduction are one of the most metabolically important processes in a multitude of diverse environments [8] including deep subsurface sediments [9, 10], caves systems [11, 12], hydrothermal vents [13, 14], microbial mats [15, 16], hypersaline waters [7, 17], or waters with extreme temperatures [18, 19]. Moreover, prokaryotic sulfide oxidation lies at the base of the food chain in several extreme habitats, as the sulfur-oxidizing bacteria mediate the energy transfer from the geothermal source to higher trophic levels, by sulfur cycling and carbon fixation [20–22].

Slovakia belongs to the Carpathian geological system with five distinct geological units with different hydrological characteristics [23]. Slovakia is extraordinarily rich in fresh groundwater [24] varying in chemical composition as a result of complex geological evolution and active tectonics of the Western Carpathians [25]. The internal part of the Western Carpathians is built by a nappe stack represented by the Tatric, Veporic, and Gemeric tectonic units, covered by thin-skinned nappe system (e.g., Fatric, Hronic) and Meliata-related tectonic units [26]. Sulfur springs examined in this study are localized in northern and western parts of Slovakia, in Central Western Carpathians, which is the mountain range rich in mineral waters, not only in the number of springs but also in a variety of compositions of mineral waters [23, 26]. One of the studied springs is localized near the Ganovce village, which is a region built of Mesozoic carbonates. Water chemistry is influenced by two mineralization processes, the dissolving of carbonates with gypsum and with anhydrite dissolution. The spring water is Ca-Mg-HCO_3_-SO_4_ type [27, 28]. The sulfur spring located near the Pastina Zavada village emerges from the Paleogene conglomerates of the Pieniny Klippen Belt, with the groundwater influenced by the flysch-type sediment [29]. Spring near the village Stankovany is one of the last active Inter-Carpathian travertine spring with high content of carbonates [30]. The out-flowing water created large travertine deposits around the well. The site represents the travertine fen fed by deep-circulation groundwater through the Mesozoic carbonates [31]. The last cold studied spring - located near Liptovske Sliace village is also localized in the Liptov basin with water associated with Holocene travertines [32]. The only thermal spring examined in this study was Scherer sulfur spring located near spa town Piestany. The area where the respective spring is located is associated with Mesozoic carbonates with the sedimentary Tatricum envelope, and the water is weakly mineralized with sulfatogenic mineralization (Ca-SO_4_-HCO_3_) [28].

In recent years, molecular studies described the microbiota of sulfur-rich thermophilic terrestrial springs [33–37]. Contrary to that, mesophilic or cold sulfur-rich waters are less studied than environments with extreme temperatures [33, 34, 38], and detailed analyses of sulfur-oxidizing mats using novel culture-independent methods are rare [39]. However, the molecular analysis provides us less biased picture of microbial diversity, the variation in DNA extraction protocols influencing the captured biodiversity is well-known (Terrat et al. 2011; Cruaud et al. 2014; Deiner et al. 2015) [40–42]. Therefore, to recover more complex information of sulfur spring bacterial diversity two different DNA extraction methods were used. Despite the richness of Slovakia in sulfur mineral waters, there are no reports on the diversity of sulfur springs in this region, except our recent studies [43–45]. Moreover, this is the first study comparing the microbiota of several sulfur springs in Slovakia using a 16 S rRNA gene sequencing. Therefore, the aim of this study was to describe the bacterial composition of five sulfur springs in Slovakia.

## Methods

### Sites description and samples collection

**Fig. 1:**
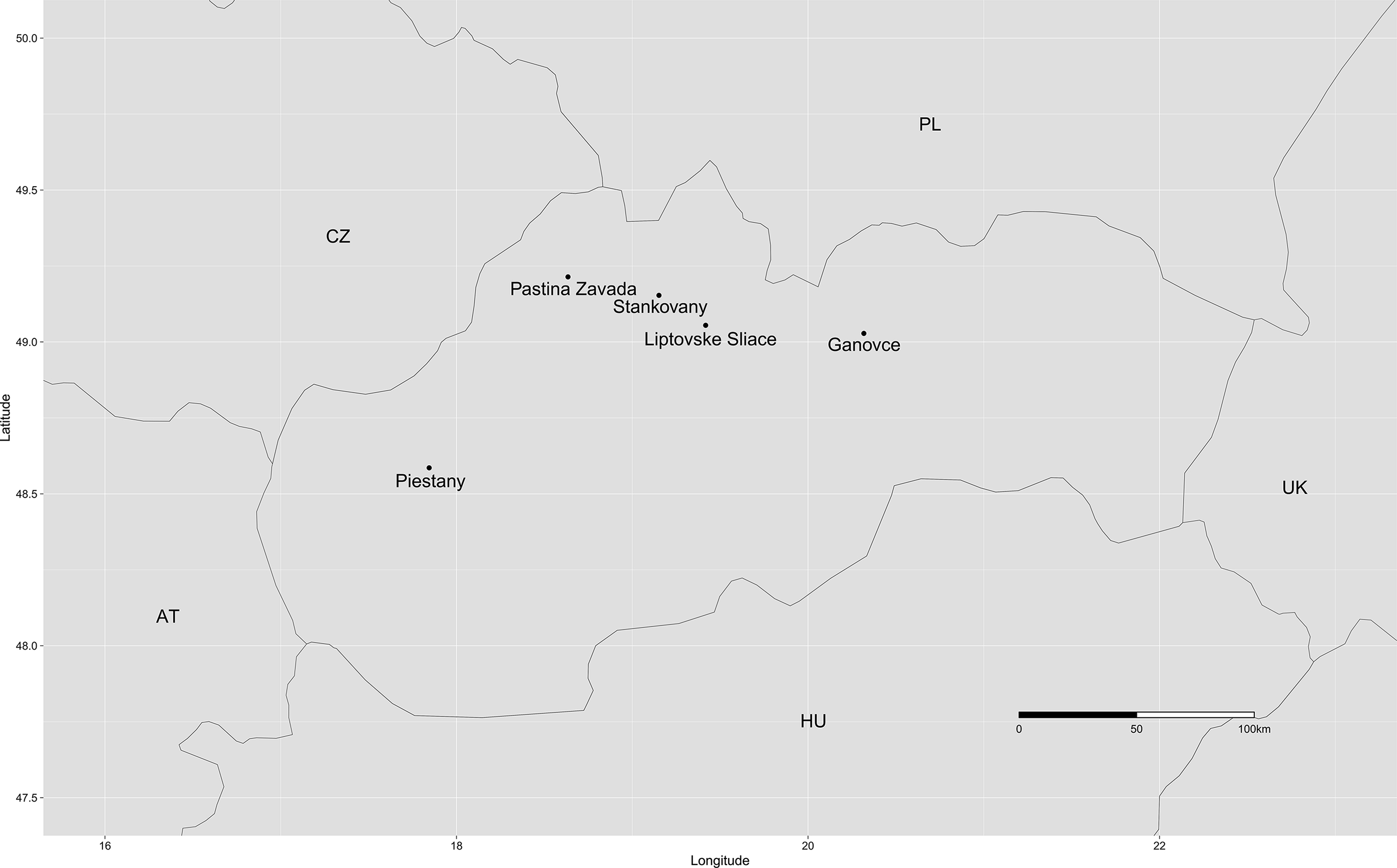
General locations of terrestrial sulfur springs where microbial mat samples were collected in this study

The microbial mat samples were collected from five sulfur springs in May 2022. The sample names were abbreviated as G, LS, PZ, P, and S corresponding to sampled sulfur springs, namely spring near a former travertine quarry in Ganovce village (G), a mineral spring situated at the northern side of a travertine hill near Liptovske Sliace village (LS), sulfur spring emerging from the borehole near the village Pastina Zavada (PZ), thermal spring Scherer emerging from the borehole in Piestany (P), spring emerging from the borehole in travertine substratum located in Stankovany (S) (Fig. 1).

Bacterial mats were collected in duplicates at the sampling points using a Pasteur pipette and placed into 1.5 mL tubes containing guanidine thiocyanate solution [100 mM Tris–HCl (pH 9.0), 4 M guanidine thiocyanate, 40 mM EDTA, and 0.001% bromothymol blue]. Microbial mat samples were frozen until the DNA was extracted. A portable Combo HI98129 multifunctional device (Hannah Instruments, USA) was used to measure the basic physiochemical parameters of springs waters directly in the fields.

### Genomic DNA extraction

The bacterial genomic DNA from microbial mat samples was extracted using two different approaches, by commercial extraction kit and by a slightly modified method described by Pospiech and Neumann [46]. This classical method consisted of the following steps. The bacterial mat samples stored in guanidine thiocyanate solution were centrifuged at 10 000 g for 10 min (Centrifuge 5420, Eppendorf, Germany), after removing the supernatant, pellets were resuspended in SET solution (0.075 M NaCl, 0.025 M EDTA, 0.02 M Tris-HCl, pH = 8). Lysozyme (Serva, Germany) and RNase A (Sigma-Aldrich, Germany) were added, and the samples were incubated for 30 min at 37 °C with constant stirring. After incubation, 1/5 of the volume of 10% sodium dodecyl sulfate and 0.5 mg/mL proteinase K (Sigma-Aldrich, Germany) were added, and samples were mixed thoroughly and incubated at 55 °C until the cells were completely lysed. Then, the cell debris was precipitated by adding 1/3 volume of 5 M NaCl. Next, one volume of chloroform was added, and the samples were incubated at laboratory temperature for 30 min with constant stirring, followed by centrifugation at 10 000 g for 10 min. The water layer was transferred into sterile tubes and the DNA was precipitated by adding 1 mL of isopropanol. After centrifugation (10 000 g for 10 min) and precipitation, supernatants were discarded, and the obtained DNA was washed with 1 mL of 70% ethanol. DNA pellets were dissolved in 50 µL of ultra-pure water. The genomic DNA from the second set of microbial mat samples was extracted employing the DNeasy PowerSoil Pro Kit (QIAGEN, Germany) as per the manufacturer’s protocol. The disintegration step was conducted on the FastPrep-24 Classic instrument (MP Biologicals, USA) device for 1 min at a maximum speed (6.5 m/s) and the obtained DNA was eluted into 60 µL of elution buffer.

Subsequently, the quality of DNA was analyzed by 1% agarose gel electrophoresis, and concentration was determined using Nanodrop OneC Microvolume UV-Vis Spectrophotometer (Thermo Scientific, USA). The DNA samples were stored at -20 °C until further use.

### Amplicons preparation and next generation sequencing

The primer pair BactB-F and BactB-R [47] and EliZyme HS FAST MIX Red (Elisabeth Pharmacon, Czech Republic) master mix were used to amplify the V4-V5 hypervariable regions of 16 S rRNA genes with the DNA extracted using two methods used as a template. Thermal cycling conditions included an initial denaturation step for 5 min at 95 °C, followed by 25 cycles of 30 s at 95 °C, 30 s at 57 °C and 30 s at 72 °C, ending with a final elongation step for 5 min at 72 °C. The quality and size of obtained PCR amplicons were evaluated on 1.5% agarose gel electrophoresis and purified using Monarch PCR & DNA Cleanup Kit (New England BioLabs, USA). Obtained amplicons were subsequently used for library preparation using the NEBNext Fast DNA Library Prep Set kit (New England Biolabs, USA) according to Milani et al. [48]. The sequencing was then performed on an Ion Torrent platform (Thermo Fisher Scientific, USA) as it was described by Mekadim et al. [49].

### Microbiome analysis

Raw partial sequences of the 16 S rRNA gene were downloaded in fastq format and analyzed using the Quantitative Insights Into Microbial Ecology (QIIME) software pipeline, version 2 (QIIME2, release 2022.2) [50] using default parameters. Briefly, raw sequences were initially imported, and the quality filtering, chimera check, and trimming were performed by the DADA2 plugin (incorporated in QIIME2) [51]. The feature table was generated, and the sequences were clustered and extracted as amplicon sequence variants (ASVs). Sequences were aligned by the MAFFT plugin [52]. Rooted and unrooted phylogenetic trees were constructed using the fasttree plugin [53]. The high-quality sequences were clustered and classified using VSEARCH against the SILVA database (version 132) with a 97% threshold [54]. The rarefaction was performed based on the sequence depth to normalize data.

Within samples, alpha diversity measures such as Shannon [55], Simpson [56], and Pielou’s evenness [57] indexes were calculated using the q2-diversity plugin based on Kruskal-Wallis test and subsequently visualized using qiime2R and ggplot2 packages in R-Studio (version 1.4.1717) [58–60]. The relative abundance of SOB in each sample was calculated and visualized as a barplot to compare the bacterial community structure of SOB between sulfur springs. Differences between samples were evaluated (beta diversity) using a multivariate statistical approach Principal Coordinate Analysis (PCoA) based on the unweighted and weighted Unifrac distance matrix [61] with communities rarefied to 7550 sequences per sample calculated by qiime2 core-metrics phylogenetic pipeline. Both UniFrax distances incorporate the phylogenetic relationships between ASVs, whilst only the weighted Unifrac matrix takes into account relative abundance. The two-dimensional PCoA plots were constructed using ggplot2 and qime2R. The influence of DNA extraction method on the bacterial community composition was evaluated by Spearman’s Rank Correlation Coefficient using PAST software (version 3.0) [62] on data of relative abundance of identified taxa.

### Nucleotide sequence data deposition

Raw partial reads of 16 S rRNA gene sequences have been deposited in the NCBI Sequence Read Archive under the BioProject accession number: PRJNA978394.

## Results

### Springs waters parameters

The pH of the water of all springs fell within the range of 5.92–7.15, however, the spring waters examined strongly differed in the TDS (Total Dissolved Solids) content. A maximum TDS value of 1998 mg/L was recorded in the S spring followed by the G spring, on the other hand, low mineralized water was in the PZ spring with content as low as 346 mg/L. The temperature of the spring waters was found to be in a range between 12.2 °C at the PZ spring to 67.5 °C at the P spring. Table 1 presents the physiochemical characteristics of water samples from five sulfur springs.

**Table 1:** Springs locations and physicochemical parameters of waters sampled

	Ganovce	Liptovske Sliace	Pastina Zavada	Piestany	Stankovany
Abbreviation	G	LS	PZ	P	S
Location (GPS coordinates)	N 49°23′24.277″ E 20°25′15.701″	N 49°3′16.81″ E 19°25′1.8″	N 49°12′25.18″ E 18°38′34.266″	N 48°35′8.192″ E 17°50′40.642″	N 49°9′15.402″ E 19°9′6.753″
pH	6.14	5.99	7.15	6.67	5.92
Temperature [°C]	22.8	20.9	12.2	67.5	19.6
TDS [mg/L]	1845	1288	346	915	1998
Conductivity	3689	2512	692	1831	3874

### 16 S rRNA gene sequencing of bacterial communities

The genomic DNA was isolated from all sulfur mats using two different extraction approaches, and the influence of the extraction technique with respect to the bacterial diversity obtained was compared. A total of 157 000 high-quality reads of 16 S rRNA gene V4-V5 variable region were obtained. Each sample consisted of 7550–24 266 reads, with a mean sequence length of 275 bp.

### Diversity analysis

**Fig. 2:**
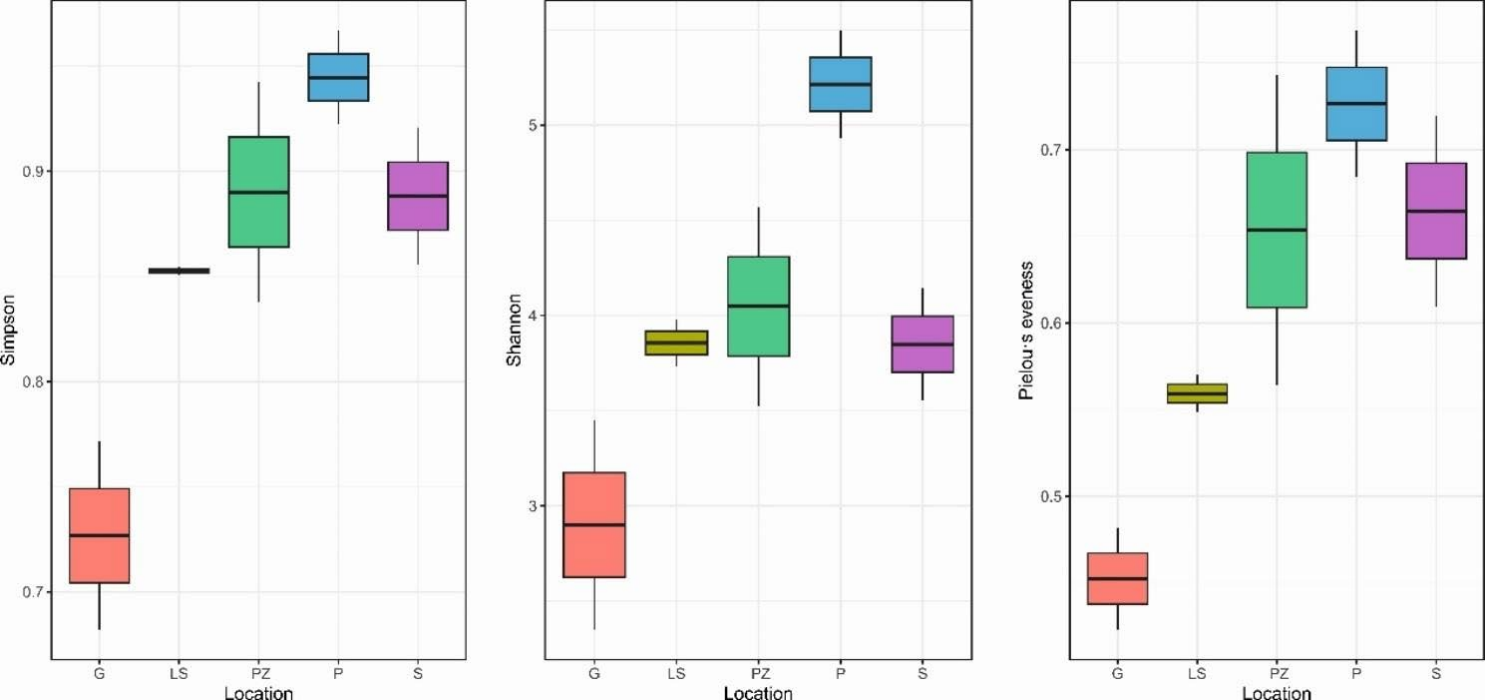
Alpha diversity indices (Simpson, Shannon, and Pielou’s evenness) of the microbiota of five studied sulfur springs. All indices showed lower diversity of the G sulfur spring microbiota

The sequences were assigned to 636 ASVs with a cutoff of 0.03. The highest number of ASVs (247) was observed in the P spring mat sample, whilst the lowest number of ASVs (87) was observed in the bacterial mat sample from the S spring. Only 4 ASVs were shared among all the studied springs, including the thermal P spring, later affiliated with *Bacillus*, *Thiothrix*, and *Brevibacterium* genera. Similarly, 4 ASVs were shared among the cold sulfur springs studied, later affiliated with *Sulfurovum*, *Sulfurispirillum*, *Thiothrix* genera, and uncultured representative of the *Halothiobacillaceae* family. Alpha diversity indices were evaluated to assess the diversity within each sample. The values of diversity indicators varied across the studied sulfur springs; however, observed differences in alpha diversity indices were below the statistical significance (Kruskal-Wallis p > 0.05). As expected, a completely different microbiota was observed in the P thermal sulfur spring. The P spring microbiota was characterized by the highest number of unique ASVs and the highest diversity assessed by all three indicators used. The lowest diversity observed by all three measures was at the G sulfur spring. The obtained results are shown in Fig. 2 as box plot graphs.

**Fig. 3:**
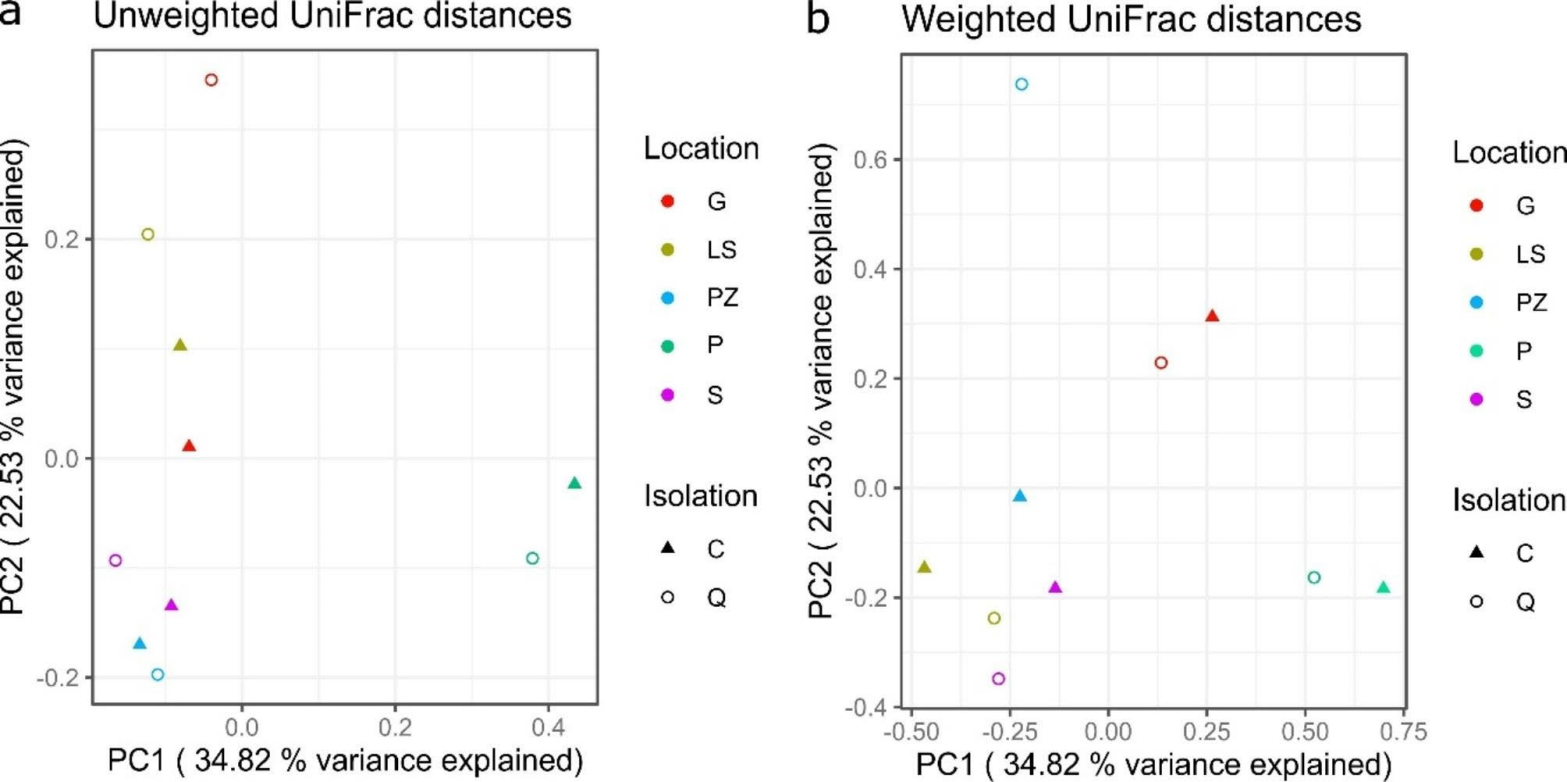
Principal coordinate analysis (PCoA) based on the unweighted (A) and weighted (B) Unifrac distance matrices of two groups of samples obtained from five sulfur springs. Each plot represents the microbiota of one sample, samples are colored based on the location. Dots represent samples with DNA extracted by classical method, whilst triangles represent samples of DNA extracted employing a commercial isolation kit. The microbiota of each spring is significantly distinct (p = 0.003, PERMANOVA with 1000 permutations)

Beta diversity indices were used to assess the variation in communities of bacterial mats among the five sulfur springs studied. Using PCoA with unweighted and weighted Unifrac distance (Fig. 3), PC1 explains 34.28% of the variation and PC2 explains 22.53% respectively. The P thermal sulfur spring presented distinct points in the PCoA, probably owing to its high temperature. Weighted and Unweighted PCoA revealed that samples generally clustered in line with the sample from the same spring. The analysis was unable to show the effect of salinity on the microbial communities. The Spearman’s Rank Correlation Coefficient showed a statistically significant correlation (p < 0.05) between bacterial mats composition of G and P sulfur springs (ranged from 0.65 to 0.78), however at phylum and class levels only (data not shown).

### Composition of bacterial community

**Fig. 4:**
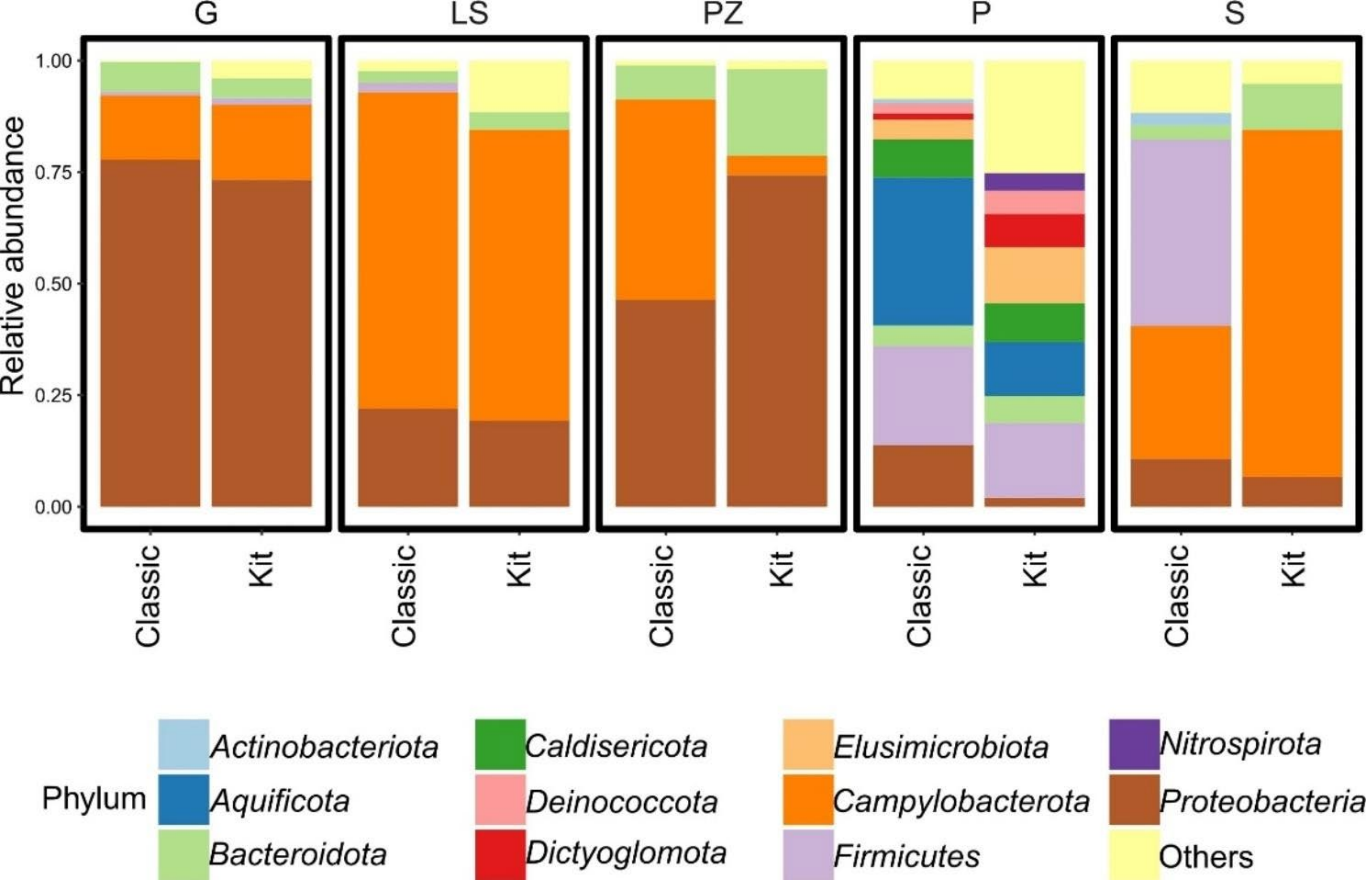
The relative abundance of bacterial phyla observed in five sulfur springs. Phyla, which represented less than 1% of identified sequences were grouped as Others. Samples of DNA extracted using two approaches were grouped based on the sulfur spring source

The taxonomic composition of each sample was estimated using the standard quality filtered sequences of 16 S rRNA gene fragments assigned with the SILVA database (version 132). The relative abundance composition of five sulfur springs showed that microbial mat samples were predominantly composed of *Proteobacteria* and *Campylobacterota* phyla, which accounted for more than 34% and 32% respectively, of all obtained sequences (Fig. 4).

Sequences affiliated with the *Proteobacteria* phylum were found to be the most abundant in most samples, except the thermal P spring sample, where their relative abundance was noticeably lower, around 8%. *Proteobacteria* counts ranged from 9% in the S spring to more than 75% in the G spring sample. The highest number of *Campylobacterota*-like sequences (68%) was observed in the LS spring samples. Conversely, the sequences affiliated with the *Campylobacterota* accounted for less than 1% in the P spring samples, where the highest abundance showed the *Aquificota* phylum with 22.6% sequences on average. Next, the *Firmicutes* phylum was the second most dominant in the P thermal spring and comprised 19.5% of obtained sequences. Moreover, *Firmicutes-*like sequences were also abundant in the S spring sample, where genomic DNA was extracted using a classical approach and accounted for 41%. The relatively abundant phylum - *Bacteroidota* accounted for almost 7% of all affiliated sequences, however, discrepancies were observed in the distribution of *Bacteroidota*-like sequences in the S spring sample. The last two phyla with an abundance higher than 1% were *Caldisericota* and *Elusimicrobiota*, though they were solely observed in the P spring.

**Fig. 5:**
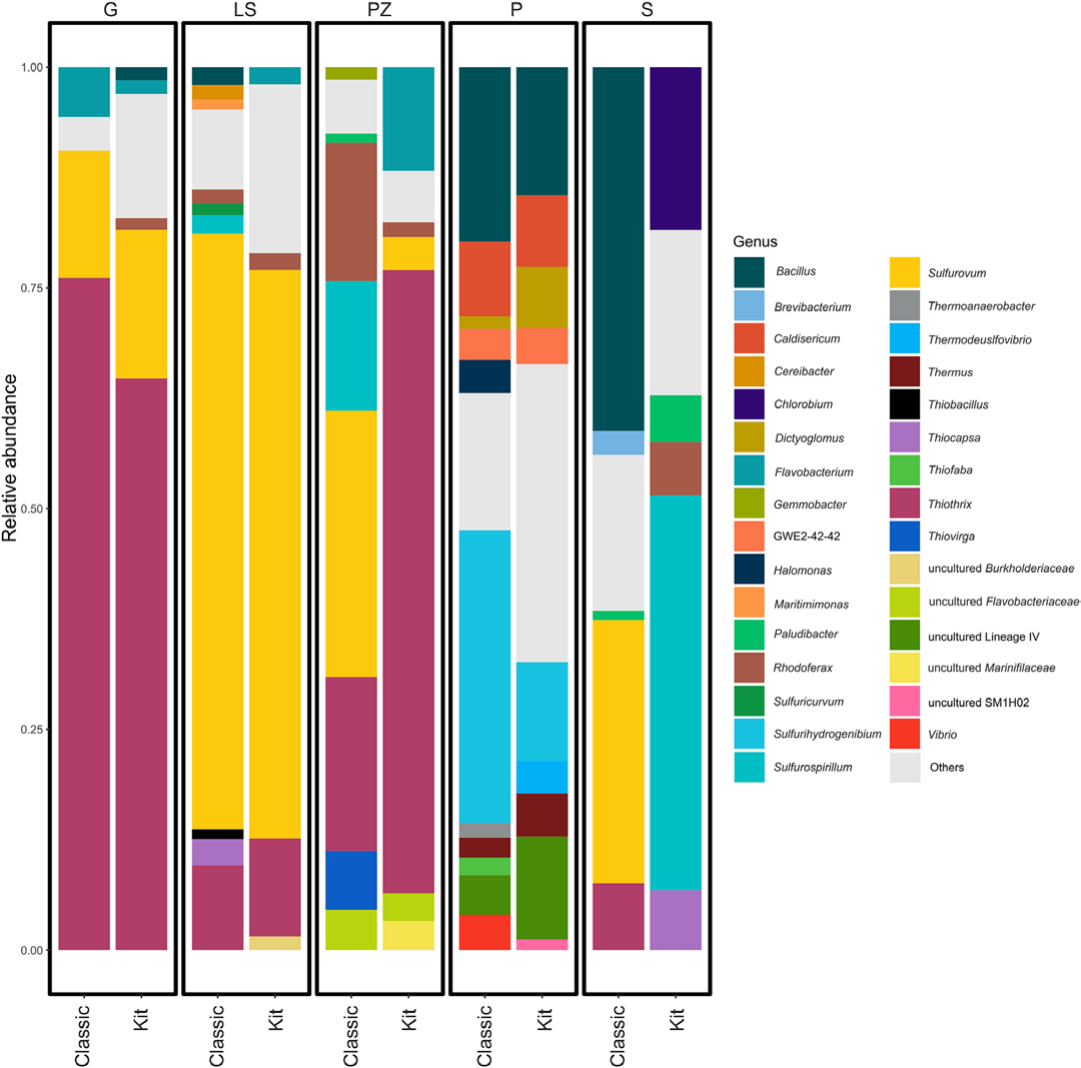
The relative abundance of bacteria genera observed in five sulfur springs. Genera, which represented less than 1% of identified sequences were grouped as Others. Samples of DNA extracted using two approaches were grouped based on the sulfur spring source

At the genus level, *Sulfurovum* was dominant and accounted for almost 30% of all bacterial populations studied. The clear dominance of this genus was observed in the microbial mats of cold LS and S spring samples, where this genus comprises 65% and 45% of all bacterial sequences. The second most abundant genus - *Thiotrix*, was identified in all samples, accounted for almost 29% of all sequences, and was the most abundant in the G spring samples using both DNA extraction approaches (76% and 64%) and one of the DNA samples from the PZ spring (70%). Despite the clear dominance of *Sulfurovum* and *Thiotrix* genera among all cold sulfur springs, their presence in the P spring samples was not detected, or the abundance was less than 1% respectively. In the P spring mat sample several taxa with relatively high abundance were observed, the most abundant was the genus *Sulfurihydrogenibium*, followed by *Bacillus*, uncultured representatives of the class *Elusimicrobia, Caldisericia*, and *Dictyoglomia*. The thermophilic genera as *Thermus*, *Thermodesulfovibrio*, *Caldisericum*, and *Dictyoglomus* were identified only in the P spring and formed a significant part of the bacterial population accounted for more than 18% sequences. The 15 most abundant genera amounted to a total relative abundance higher than 80% of the prokaryotic community, and the genera with a relative abundance higher than 1% at least in one sample are shown in Fig. 5.

### Sulfur-oxidizing bacteria

**Fig. 6:**
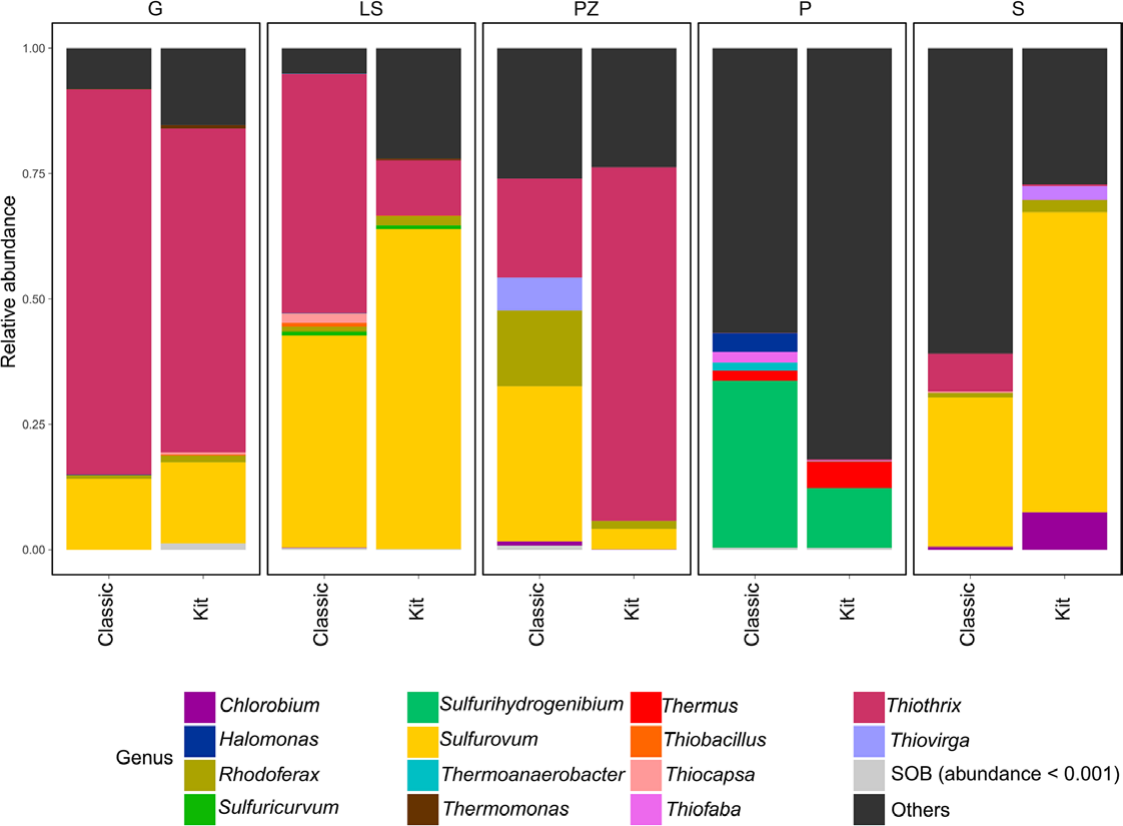
The relative abundance of identified sulfur-oxidizing genera. Sulfur-oxidizing genera with a relative abundance lower than 0.001 were grouped as SOB. Non-sulfur-oxidizing genera were grouped as Others. Samples of DNA extracted using two approaches were grouped based on the sulfur spring source

All the detected sulfur-oxidizing bacteria belonged to one of six bacterial phyla, from which most representatives were affiliated with the *Proteobacteria* phylum (~ 31%). Among the genera identified, 33 could be considered sulfur-oxidizing, from which genera *Thiothrix* and *Sulfurovum* were dominant (Fig. 6). Altogether, 66% of sequences obtained from sulfur springs mat samples were affiliated with one of the sulfur-oxidizing genera. The highest prevalence of sulfur-oxidizing bacterial population was recorded in G sulfur spring where sulfur-oxidizing bacteria-related sequences accounted on average for 88% of the total eubacterial population (~ 91% in DNA samples extracted by classical approach, ~ 84% in DNA samples extracted using kit). In terms of the abundance of sulfur-oxidizing microorganisms, the G spring was followed by the LS spring (~ 85% in DNA samples extracted by classical approach, ~ 78% in DNA samples extracted using kit), the PZ spring (~ 73% in DNA samples extracted by classical approach, ~ 76% in DNA samples extracted using kit), and the S spring (~ 39% in DNA samples extracted by classical approach, ~ 72% in DNA samples extracted using kit). In addition to the clear dominance of the *Thiotrix* and *Sulfurovum* genera, the *Rhodoferax* genus was identified in all cold sulfur springs samples. Other relatively abundant genera were *Chlorobium* (7.4%) in the S sulfur spring sample of DNA extracted using kit, *Thiovirga* (6.6%) in the PZ spring sample of DNA extracted using classical approach and *Thermus* (5.2%) in the P sulfur spring sample of DNA extracted by kit. The lowest number of sulfur-oxidizing bacteria observed was in the P spring, where the sequences affiliated with sulfur-oxidizing microorganisms accounted for only approximately one-third of obtained sequences. Sulfur-oxidizing genera with a relative abundance higher than 1% are shown in Fig. 6.

## Discussion

The territory of Slovakia is formed by the Western Carpathians whose present-day geological structure is generally a result of the Alpine orogenic stage. Due to the existence of five distinct geological units with different hydrological characteristics the Slovakia territory is extraordinarily rich in fresh groundwater [23, 24] varying in chemical composition resulting from geological evolution and active tectonics of the Western Carpathians [25]. Most springs outflow from the Inner Carpathian depression, at the marginal faults between the mountains and lowlands [63]. The chemical composition of mineral water depends mainly on the aquifer rock. The main sulfur sources in the water of the Western Carpathians are sediments of the Permian, Triassic, and Tertiary ages [64]. According to the assessed temperature G, LS, and S springs may be considered mesothermal, and the PZ as a cold sulfur spring. The P spring with a temperature of 67.5 °C could be considered a thermal sulfur spring as the temperature of the water is considerably higher than annual air temperature at that location. Microbial populations inhabiting mesothermal and cold terrestrial sulfur springs are less studied compared to other freshwater environments, and recently it was shown, that these environments harbor unique microbial communities, as a reflection of the nature of upwelling groundwaters [65]. On the contrary, thermal sulfur springs are a source of well-adapted microbiota considered a model of ancient life, and bacteria with the ability to produce thermostable enzymes [66, 67]. Based on the salinity of the spring water these five springs can be categorized into three groups [68]: high salinity (S, G), medium salinity (LS, P), and low salinity (PZ). In all studied springs, white microbial mats and filaments were observed indicating the presence of sulfur-oxidizing bacteria.

During the characterization of five sulfur springs microbiota, 24 bacterial phyla were identified using a 16 S rRNA gene sequencing, ten of which were most abundant and accounted for more than 90% of the microbial population (Fig. 4). Sequencing analysis showed that the phyla *Proteobacteria* and *Campylobacterota* were dominant in almost all samples, which is in accordance with the previous reports about the *Proteobacteria* phylum dominating bacterial communities of sulfur environments [39, 65, 69]. Similarly, the *Campylobacterota* phylum (known also as *Epsilonbacteraeota*), has been found to be a significant part of different sulfur-rich environments [33, 70–72]. Moreover, according to Campbell et al. [73], representatives of *Campylobacterota* are a key part of sulfidic environments. The *Firmicutes* phylum was relatively abundant (16–41%), especially in the S and P springs, which is in agreement with the results reported by Perreault et al. [74] and Sharma et al. [75], where the phylum represented ~ 20% of the bacterial population of sulfur-rich or thermal waters. However, the abundance of the *Firmicutes* phylum in DNA samples obtained from the S spring strongly differed (< 1% and more than 40% regarding the DNA extraction technique used). Representatives of the *Bacteroidetes* phylum were identified in all samples, however, the abundance was relatively low, only 6% on average. Similar results were reported by Perreault et al. [76] on the microbiota of sulfur springs, as well as other studies on freshwater environments [66, 77]. The most distinct microbial community was observed in the thermal P spring, as members of *Aquificota, Caldisericota*, *Elusimicrobiota*, *Dictyoglomota*, and *Deinococcota* phyla were not observed in other springs. From those, *Dictyoglomota, Deinococcota*, and *Aquificota*, represented by one genus solely, are considered a typical part of hot spring microbiota [34, 78].

The sulfur-oxidizing bacteria dominated the microbiota of studied mats, as the sequences of sulfur-oxidizing genera accounted for 30–88%. The *Thiothrix* genus was reported in each sampling site, and it accounted for up to 70% of the bacterial community in the G spring. Although its maximum growth temperature is around 35 °C [79], representatives of this genus were observed in the P spring (~ 67 °C) as well. The bacterial genus *Thiothrix* is frequently encountered in sulfidic springs [80, 81]. Interestingly, another *Gammaproteobacteria* genus - *Thiofaba* was also identified in the thermal P spring and accounted for 2% of sequences of DNA samples extracted by classical approach, despite the maximum growth temperature of 51 °C [82]. Contrary to that, the mesophilic bacterial genus *Thiovirga* [83] comprised 6% of sequences from the PZ spring of DNA extracted by classical approach, where the assessed temperature was 12 °C. To the best of our knowledge, this is the first evidence of the *Thiovirga* genus constituting a significant part of the bacterial community of cold terrestrial sulfidic springs. Additionally, green and purple sulfur-oxidizing genera *Chlorobium* (~ 0.9%) and *Thiocapsa* (~ 0.6%) were relatively abundant considering their average frequencies. In addition to the *Thiothrix* genus as a key part of the microbiota of terrestrial sulfur springs in Slovakia, almost 30% of obtained sequences were affiliated with the genus *Sulfurovum*, implying the presence of a relatively stable bacterial community. A clear prevalence of this was observed in the LS spring, where it comprised 60% of the bacterial population.

Among the five sulfur springs studied, genera *Thiothrix* and *Sulfurovum* constituted the majority of obtained sequences. *Thiothrix* genus was identified using a noncultivation approach among other cold sulfur springs in Slovakia [44, 45]. Contrarily, this is the first evidence of the *Sulfurovum* genus constituting a large part of the microbiota of Slovak sulfur springs. Several studies suggested the *Sulfurovum* genus represents the primary producer of different sulfur-rich environments, similarly, the *Thiothrix* species are considered to be a typical part of sulfur-oxidizing microbial communities in sulfur-rich habitats, probably due to their potential to colonize a geochemically wider range of environments [70, 71, 84]. Generally, *Thiothrix* species tend to colonize oxygenated habitats with lower sulfide levels in cold sulfur springs. The deeper areas of sulfur springs are likely anaerobic and despite that sulfide concentration may be higher, the unavailability of oxygen restricts the *Thiothrix* species to the upper parts of sulfur springs and to filaments [81]. Conversely, *Sulfurovum* species tend to be more abundant closer to the source of reduced sulfur with a lower concentration of oxygen [71, 85]. Sulfur-oxidizing *Campylobacterota* representatives are generally unable to store sulfur intracellularly, a characteristic that may have important implications for the sulfur cycle and which may limit their ability to consume toxic levels of oxygen in the absence of high sulfide concentrations. Contrary to that, *Thiothrix* species store sulfur intracellularly, thus allowing them to thrive also in environments with the limiting availability of sulfide [71, 81, 85]. Patwardhan et al. [86] observed that *Sulfurovum* species are more abundant in young filaments. They may be dominant pioneers, and after the primary filaments are established other bacteria such as sulfur-oxidizing *Gammaproteobacteria* may appear [86]. Correspondingly, abundant *Sulfurovum*-related species accompanied by the abundant *Thiothrix* species were observed in various sulfur-rich environments [38, 81].

The abundance and diversity results were obtained through the analysis of amplicon sequencing data. The highest diversity observed was in the thermal P spring which is documented in Fig. 2. The S, PZ, and LS springs showed similar levels of diversity, while the lowest diversity was observed in the G spring due to the predominance of a single genus–*Thiothrix* (~ 70%) in this spring. Also, PCoA analysis showed the relatedness of these samples (Fig. 3), which may be due to similar environmental parameters. The P thermal sulfur spring clustered separately, with a distinctive diverse microbiota, probably due to higher water temperature.

Various environmental parameters may introduce bias during the metagenomic DNA extraction process. The effect of the DNA extraction method on bacterial diversity has been examined in various environments [87]. According to Spearman’s Rank Correlation Coefficient, there was a strong correlation between microbiota composition at the phylum and class levels of two DNA extraction methods of the G and the P springs respectively. For the rest of the springs, no statistically significant correlation between samples was observed, indicating the influence of the DNA extraction method used onto the observed microbial composition at different taxonomic levels. Moreover, PCoA analysis did not show a clear influence of the extraction method used on the diversity of sulfur spring microbiota. Nevertheless, the influence on the relative frequency of several taxa was observed. Bacterial community composition of the S spring was affected by the DNA extraction protocol. The abundance of the *Firmicutes* phylum was reduced from 40% of the bacterial population to 0.2% using the commercial DNA isolation kit. A similar result was observed in the thermal P spring, where usage of this isolation approach reduced the *Aquificota* phylum by 40%. Differences in the abundance of another phylum *Campylobacterota* were observed in the P and S springs. A classical DNA isolation approach decreased the abundance of this phylum from 77 to 30% in the S spring. On the other hand, we observed an increase in the same phylum in the spring PZ. Within them, the most remarkable difference was observed in the abundance of the genus *Sulfurovum* in the P and S springs, respectively.

## Conclusion

The understanding of sulfur-oxidizing microbiota living in cold sulfur springs is still limited. By a 16 S rRNA gene sequencing employing two DNA extraction methods, five sulfur springs microbiota was compared. Our results emphasize the diversity of unique cold sulfur springs microbiota. The core microbial taxa were identified, and the biogeochemical importance and ecological success of classes *Gammaproteobacteria* and *Campylobacteria* classes was confirmed. Moreover, our study indicated that the *Sulfurvoum* and *Thiothrix* are key players of sulfur cycles in cold but not hot sulfur springs. However, further experiments need to be carried out, as the only one hot sulfur spring microbiota was studied and to elucidate the influence of the DNA extraction method on the observed bacterial diversity.

## Data Availability

The datasets of the raw partial reads of 16 S rRNA gene sequences generated and analysed during the current study are available in the NCBI Sequence Read Archive under the BioProject accession number: PRJNA978394.
